# Prevalence and incidence of mastocytosis in adults: a Danish nationwide register study

**DOI:** 10.1007/s10654-024-01195-5

**Published:** 2025-01-03

**Authors:** Maren Poulsgaard Jørgensen, Andreas Kiesbye Øvlisen, Jonas Faartoft Jensen, Tarec Christoffer El-Galaly, Maiken Glud Dalager, Hanne Vestergaard, Sigurd Broesby-Olsen, Marianne Tang Severinsen

**Affiliations:** 1 Department of Hematology, Clinical Cancer Research Center, Aalborg University Hospital, Aalborg, Denmark; 2 Department of Clinical Medicine, Aalborg University, Aalborg, Denmark; 3 Department of Hematology, Aarhus University Hospital, Aarhus, Denmark; 4 Department of Dermatology, Aalborg University Hospital, Aalborg, Denmark; 5 Department of Hematology, Odense University Hospital, Odense, Denmark; 6 Mastocytosis Centre, Odense University Hospital (MastOUH), Odense University Hospital, Odense, Denmark; 7 Department of Dermatology and Allergy Centre, Odense University Hospital, Odense, Denmark

**Keywords:** Mastocytosis, Prevalence, Incidence, Overall survival, Population-based

## Abstract

Mastocytosis is a group of rare heterogeneous diseases with a prevalence previously found to be 10–23 per 100,000 persons. More awareness and improvements in the diagnostic methods in later years have led to more patients being diagnosed. Here, we set out to present the prevalence and incidence rate of mastocytosis among the adult Danish population. By merging data from the Danish National Patient Register, the Danish Pathology Register and the Danish Cancer Register we included all adult patients (≥ 18 years) diagnosed with mastocytosis in Denmark prior to 2022. A cohort of 1,594 patients with mastocytosis was identified. The prevalence of mastocytosis was 27.43 per 100,000 persons (95% confidence interval [CI]: 25.95–28.96) as of January 1, 2022, and the 25-year average incidence rate between 1997 and 2021 was 1.21 per 100,000 persons (95%CI: 1.02–1.40) with an increasing incidence rate since 2002. We found a higher prevalence of mastocytosis among adults in the Danish population than previously reported, and an increasing incidence rate during the last 20 years. Increased awareness of the disease and better diagnostic methods most likely contributed to this.

**Supplementary Information:**

The online version contains supplementary material available at 10.1007/s10654-024-01195-5.

## Introduction

Mastocytosis is a group of rare heterogeneous diseases in which abnormal mast cells proliferate and accumulate most often in the skin, bone marrow (BM), and gastrointestinal tract [1–3]. Mastocytosis is categorized as cutaneous mastocytosis (CM) and systemic mastocytosis (SM) according to the involved organs. Cutaneous mastocytosis, which is limited to the skin, is mostly found among children and spontaneous regression often occurs in puberty. By contrast, SM is a chronic disease with signs and symptoms from extracutaneous organs [3]. The existing epidemiologic literature on SM is limited by the rarity of the disease and lack of awareness leading to the diagnosis of SM being overlooked or delayed for years [4, 5]. Diagnosis of SM is based on major and minor criteria defined by the World Health Organization (WHO) (Table 1) [6–9]. Adult patients with typical skin lesions but no BM biopsy performed yet are provisionally classified as mastocytosis in the skin (MIS) [7, 10]. Previous studies have shown that most will have SM when fully examined [10, 11]. According to the WHO, SM is divided into non-advanced i.e., indolent SM (ISM), bone marrow mastocytosis, and smoldering SM or advanced SM (AdvSM) i.e., aggressive SM (ASM), SM with an associated hematologic neoplasm (SM-AHN), and mast cell leukemia (MCL) [7, 9, 12]. In 2022, the International Consensus Classification (ICC) adjusted the classification of SM-AHN to solely involve patients with SM with an associated myeloid neoplasm (SM-AMN) [13], whereas the WHO classification remained unchanged [8]. Life expectancy for patients with non-advanced SM is comparable to the general population whereas patients with AdvSM have significantly lower survival rates [14, 15].

**Table 1:** Diagnostic criteria for systemic mastocytosis

**Major criterion**
Multifocal, dense infiltrates of mast cells (≥ 15 mast cells) in the BM and/or extracutaneous organs
**Minor criteria**
≥ 25% atypical mast cells on BM smears or ≥ 25% spindle-shaped mast cell in infiltrates in BM or other extracutaneous organs cKIT mutation in D816V or in other critical regions of KIT in blood, BM or extracutaneous organ Mast cells in BM, blood or extracutaneous organ express CD25 and/or CD2 and/or CD30 in addition to normal mast cell markers Baseline serum tryptase > 20 ng/ml persistently
**Diagnostic criteria**: either 1 major and 1 minor criterion or 3 minor criteria

World Health Organization diagnostic criteria for systemic mastocytosis.

BM: Bone marrow. Adapted from Pardanani et al. 2023.

Previously, the prevalence of mastocytosis have been found to be 10–13 per 100,000 adult persons [5, 16–18]. A higher prevalence of 23.9 per 100,000 adult persons was found in a recent Swedish register study [19]. Over the past decades much effort has been put into increased awareness of mastocytosis internationally [20, 21] as well as in Denmark with the establishment of a national, multidisciplinary Centre of Excellence in 2010 (Mastocytosis Centre Odense University Hospital). New diagnostic tools such as sensitive analysis of KIT D816V mutation in peripheral blood have been developed and become easily accessible [22–25]. These efforts make it likely that more patients have been diagnosed during the last decade. Furthermore, new treatment options and the use of new prognostic scoring tools to aid treatment strategies may have affected survival in patients with AdvSM [22, 26, 27].

In this nationwide population-based register study we set out to examine the present prevalence and incidence rate of mastocytosis among the adult Danish population during a 25-year period from 1997 to 2021 and assess the overall survival (OS) of Danish patients with mastocytosis compared with the general population.

## Methods

### Setting

This nationwide cohort study was conducted in Denmark with a study period from January 1, 1997, to December 31, 2021. The Danish population is registered in the Danish Civil Registration System (CRS) established in 1968. The CRS holds information on the entire Danish population including name, sex, date of birth, citizenship, immigration, emigration, vital status, and eventually date of death. The information in the CRS is linked to the unique 10-digit Central Personal Register number assigned to all residents upon birth or immigration [28–30]. Use of health services are registered in nationwide registers using this number which enables complete and long-term follow-up on an individual level [31, 32]. The patient population in this study was constructed by merging data from the Danish National Patient Register (NPR), the Danish Pathology Register (DPR) and the Danish Cancer Register.

### Registers

The NPR is administered by the Danish Health Authority and contains information on all inpatient somatic admissions and outpatient visits since 1977 and 1995, respectively, including diagnosis codes, date, and department of contact. Diagnoses were classified according to the International Classification of Diseases (ICD), revision 8 (ICD-8) prior to 1994, and afterwards ICD, 10th revision (ICD-10) [32]. In this study, the NPR was used to identify potential patients with mastocytosis (supplementary Table S1). To further identify patients, validate the diagnosis, and distinguish between subtypes information from the DPR was used.

The DPR was established in 1997 by the Danish National Board of Health and holds information on all biopsies and pathological examinations conducted in Denmark including historical pathology data going back to the 1970s. The information in the register is coded using a Danish version of the Systematized Nomenclature of Medicine (SNOMED) [33, 34]. In this study, the SNOMED codes for mastocytosis were paired to the topography codes for the individual biopsies to distinguish between cutaneous and extracutaneous biopsies (supplementary Table S2).

The Danish Cancer Register was used as it holds information on all incidences of cancer in Denmark since 1943 [35, 36]. In this register patients were identified using ICD-10 codes.

Information on KIT mutational status was collected from the DPR and the Register of Laboratory Results for Research (RLRR) which was also used to examine the tryptase levels. Data on KIT mutational status was gradually implemented in the DPR during the 2010s. The RLRR is a nationwide register of laboratory results including the Nomenclature for Properties and Units code and date of sampling. The RLRR has varying coverage as data collecting began at different times; however, the register is considered complete since 2015 [31, 37].

### Study population

The patient cohort include all adult (≥ 18 years) patients diagnosed with mastocytosis in Denmark prior to 2022, who were either alive or born after 1997 and had Danish residency at the time of diagnosis. A diagnosis of mastocytosis was considered valid if either of the following criteria were met: (1) biopsy-verified mastocytosis, (2) two or more ICD codes over time for mastocytosis given by a dermatologist, hematologist, or an allergist, or (3) a single ICD code for mastocytosis given by a dermatologist, hematologist, or an allergist supplemented with a positive KIT mutation and/or high tryptase levels ≥ 20 ng/ml. Patients solely classified as *D47.0 Histiocytic and mast cell tumors of uncertain and unknown behavior* with no positive diagnostic tests for mastocytosis were excluded as further inspection showed that most of these patients had biopsy-verified histiocytosis. Inclusion date was defined as the first date of any mastocytosis diagnosis across the registries. For patients with childhood mastocytosis that continued into adulthood (determined by a biopsy or ICD codes registered after the age of 18), inclusion was the date of their 18th birthday. Patients with SM were categorized according to the most severe WHO subtype [7, 9] recorded; however, as it was not possible to subcategorize non-advanced SM into bone marrow mastocytosis, smoldering mastocytosis, or ISM, all patients with non-advanced SM were characterized as ISM. A diagnostic delay of 180 days was implemented in relation to progression to AdvSM i.e., patients that progressed during this time were considered as having AdvSM from the start. Patients with only a positive skin biopsy were assessed on the presence of a BM biopsy in the DPR. Patients without pathologic findings in the BM biopsy were classified as CM and patients who did not have a BM performed were classified as MIS. If another hematologic disease was found in the BM biopsy, the patient was classified as SM-AHN [8, 38]. The AHNs were grouped as myelodysplastic syndrome (MDS), myeloproliferative neoplasm (MPN) (polycythemia vera, essential thrombocythemia, primary myelofibrosis, chronic myeloid leukemia), MDS/MPN unclassifiable (MDS/MPN-U) (e.g., chronic myelomonocytic leukemia), acute leukemia, and lymphoproliferative disorder (lymphoma, multiple myeloma, chronic lymphocytic leukemia). Patients were categorized with more than one group of AHN if verified across the registries.

### Statistical analyses

Baseline characteristics were presented as medians with interquartile range (IQR) for continuous variables and as proportions for categorical variables. Test of differences in distributions between subgroups within continuous and categorical variables was conducted using Wilcoxon rank sum tests and $\:{\chi\:}^{2}$-tests, respectively. Due to data protection, values for groups < 3 and groups that can be used to identify groups < 3 were censored by clustering the advanced subgroups as AdvSM.

Prevalence of mastocytosis was calculated as of January 1, 2022. The most severe subtype of mastocytosis reached at the end of the study was registered. Incidence rates were calculated from 1997 to 2021 according to the initial mastocytosis subtype diagnosed. The average incidence rate was calculated based on the mean population in Denmark (≥ 18 years) each year from 1997 to 2021.

Overall survival was estimated using the Kaplan-Meier estimator with OS defined as time from initial diagnosis to death (from any cause) or censoring (end of follow-up on December 31, 2022, or emigration) whichever came first. The survival of patients with mastocytosis was compared to the general population by matching each patient on sex and birthyear with five persons from the general Danish population without a prior diagnosis of mastocytosis at the time of inclusion of the index patient. Differences in OS were tested using restricted mean survival differences at 20 years after diagnosis. The 10-year survival probability was presented for each subtype of mastocytosis, and the median survival was presented for subtypes with high mortality. Patients who had childhood-onset mastocytosis were not included in the survival analyses. Median follow-up was calculated using the reverse Kaplan-Meier method. To compare OS between the different subgroups of mastocytosis within the first 10 years of follow-up, hazard-ratios (HRs) were calculated using a Cox proportional hazards model. These HRs were adjusted for sex, age at initial diagnosis, and year of initial diagnosis using inverse probability weights estimated using a multinomial logistic regression model. The assumption of proportional hazards was confirmed by visual inspection of Schoenfeld residuals.

Statistical analyses were performed using SAS version 9.4 (SAS Institute Inc, Gary, North Carolina, USA) and R statistical software (version 4.2.2, R Foundation for Statistical Computing, Vienna, Austria). P-values of < 0.05 were considered statistically significant. The project was approved by the Region North, Denmark (F2022-009).

## Results

### Mastocytosis cohort

**Fig. 1:**
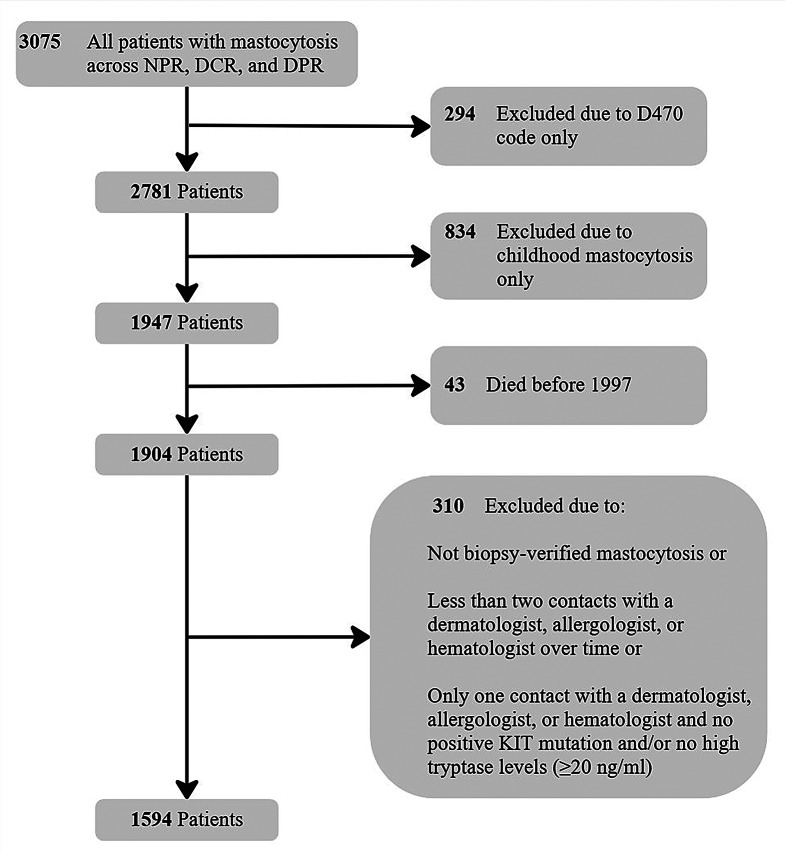
Flowchart of cohort identification with specification of exclusion criteria

A total of 1,594 adult patients with mastocytosis were identified in the Danish population (Fig. 1). Most were women (57.6%) with a median age of 49 years (IQR: 37–63 years) at initial diagnosis. The most prevalent subgroup was MIS (780, 48.9%) followed by ISM (488, 30.6%), SM-AHN (176, 11.0%), CM (132, 8.3%), ASM (10, 0.6%), and MCL (8, 0.5%). Using the ICC classification the subgroups was: MIS 791 (49.6%), ISM 522 (32.7%), AdvSM 149 (9.3%), and CM 132 (8.3%). Patients with AdvSM were generally older (67 years, IQR: 58–74 years) and more likely to be male (54.6%). Most patients with SM had a positive KIT mutation (ISM: 95.8%, AdvSM: 84.0%) and a high tryptase level (ISM: 63.2%, AdvSM: 69.6%). The median tryptase level across the entire cohort was 20 ng/ml (IQR: 9–45 ng/ml) but were considerably higher among patients with MCL (247 ng/ml [IQR:171–322 ng/ml]) (Table 2).

**Table 2:** Characteristics of patients with mastocytosis by subtype, prior to 2022

Characteristic	Overall	CM	MIS	ISM	ASM	SM-AHN	MCL	AdvSM
Total, *n*(%)*	1,594 (100%)	132 (8.3%)	780 (48.9%)	488 (30.6%)	10 (0.6%)	176 (11.0%)	8 (0.5%)	194 (12.2%)
**Age**,** median (IQR)**	49 (37–63)	48 (38–60)	45 (32–58)	49 (40–63)	52 (49–68)	67 (59–75)	67 (64–70)	66 (58–74)
**Age group**, n(%)**								
18–40 41–60 > 60	493 (30.9%) 643 (40.3%) 458 (28.7%)	39 (29.5%) 60 (45.5%) 33 (25.0%)	315 (40.4%) 305 (39.1%) 160 (20.5%)	129 (26.4%) 224 (45.9%) 135 (27.7%)	… … …	… … …	… … …	10 (5.2%) 54 (27.8%) 130 (67.0%)
**Sex**,** n(%)**								
Female Male	918 (57.6%) 676 (42.4%)	84 (63.6%) 48 (36.4%)	483 (61.9%) 297 (38.1%)	263 (53.9%) 225 (46.1%)	7 (70.0%) 3 (30.0%)	78 (44.3%) 98 (55.7%)	3 (37.5%) 5 (62.5%)	88 (45.4%) 106 (54.6%)
**Year of initial diagnosis**,** n(%)**								
< 1997 1997–2001 2002–2006 2007–2011 2012–2016 2017–2021	233 (14.6%) 157 (9.8%) 163 (10.2%) 265 (16.6%) 336 (21.1%) 440 (27.6%)	16 (12.1%) 13 (9.8%) 9 (6.8%) 28 (21.2%) 22 (16.7%) 44 (33.3%)	171 (21.9%) 102 (13.1%) 96 (12.3%) 136 (17.4%) 130 (16.7%) 145 (18.6%)	27 (5.5%) 23 (4.7%) 35 (7.2%) 80 (16.4%) 134 (27.5%) 189 (38.7%)	… … … … … …	… … … … … …	… … … … … …	19 (9.8%) 19 (9.8%) 23 (11.9%) 21 (10.8%) 50 (25.8%) 62 (32.0%)
**Death status at end of 2021**,** n(%)**								
Alive Dead	1,295 (81.2%) 299 (18.8%)	108 (81.8%) 24 (18.2%)	658 (84.4%) 122 (15.6%)	442 (90.6%) 46 (9.4%)	6 (60.0%) 4 (40.0%)	81 (46.0%) 95 (54.0%)	0 (0.0%) 8 (100.0%)	87 (44.8%) 107 (55.2%)
**Type of diagnostic biopsy**,** n(%)**								
BM + skin BM alone Skin alone Other extracutaneous No biopsy	265 (16.6%) 362 (22.7%) 798 (50.1%) 24 (1.5%) 145 (9.1%)	0 (0.0%) 0 (0.0%) 75 (56.8%) 0 (0.0%) 57 (43.2%)	0 (0.0%) 0 (0.0%) 695 (89.1%) 0 (0.0%) 85 (10.9%)	236 (48.4%) 228 (46.7%) 0 (0.0%) 24 (4.9%) 0 (0.0%)	… … … … …	… … … … …	… … … … …	29 (14.9%) 134 (69.1%) 28 (14.4%) 0 (0.0%) 3 (1.5%)
**KIT test**,** n(%)**								
Negative Positive	142 (19.0%) 607 (81.0%)	48 (57.1%) 36 (42.9%)	59 (44.4%) 74 (55.6%)	18 (4.2%) 408 (95.8%)	… …	… …	… …	17 (16.0%) 89 (84.0%)
Missing information	845	48	647	62	…	…	…	88
**Tryptase ≥ 20 ng/ml measured**,** n(%)**								
Yes No	449 (56.3%) 349 (43.7%)	28 (35.4%) 51 (64.6%)	75 (41.2%) 107 (58.8%)	275 (63.2%) 160 (36.8%)	… …	… …	… …	71 (69.6%) 31 (30.4%)
Missing information	796	53	598	53	…	…	…	92
**Tryptase level (ng/ml)**,** median (IQR)**	20 (9–45)	9 (5–22)	12 (6–22)	24 (12–55)	52 (29–93)	30 (11–65)	247 (171–322)	34 (13–76)

IQR: interquartile range, CM: Cutaneous mastocytosis, MIS: mastocytosis in the skin, ISM: indolent systemic mastocytosis, ASM: aggressive systemic mastocytosis, SM-AHN: systemic mastocytosis with associated hematologic neoplasm, MCL: mast cell leukemia, AdvSM: ASM, SM-AHN, and MCL. Values for groups < 3 and groups that could be used to identify groups < 3 are censored

* Diagnosis registered at end of study on December 31st, 2021, at emigration or at death

** Age at initial diagnosis or inclusion. Patients with childhood mastocytosis that continued into adulthood were included at the age of 18 years

During the study period 52 patients progressed after the diagnostic delay. 48 patients (3.6%) progressed from an initial non-advanced form (ISM: 29 patients [5.6%], MIS: 19 patients [2.3%]) to an AdvSM and four patients changed from one subtype of AdvSM to another (i.e. either from ASM to SM-AHN, ASM to MCL, or SM-AHN to MCL). In total, five patients progressed to ASM, four to MCL, and 43 to SM-AHN. The non-advanced patients that progressed were older at the time of diagnosis (ISM: 56 years (46–70 years), MIS: 59 years (46–64 years)) compared to patients that did not progress (*p* = 0.012 and *p* = 0.008, respectively) and slightly more often female (54.2%). Before progression patients with an initial diagnosis of ISM had a median tryptase level of 49 ng/ml (IQR: 40–71 ng/ml) compared to 22 ng/ml (IQR: 11–52 ng/ml) among ISM patients that did not progress (*p* = 0.04) (supplementary Table S2). Median time from diagnosis to progression was 7.0 years (IQR: 2.4–13.5 years) among patients with non-advanced mastocytosis. Following the ICC classification only 29 non-advanced patients (2.2%) progressed during the study period (ISM: 20 patients (3.7%), MIS: 9 patients (1.1%).

In this study, most of the hematologic neoplasms associated with mastocytosis were of myeloid lineage. Patients with SM-MPN amounted to 47 patients (26.7%), SM-acute leukemia to 37 patients (21.0%), SM-MDS/MPN-U to 35 patients (19.9% of which 25 patients had chronic myelomonocytic leukemia), and SM-MDS to 25 patients (14.2%). 51 patients (29.0%) had an associated hematologic neoplasm of lymphoid lineage.

### Epidemiology of mastocytosis

**Fig. 2:**
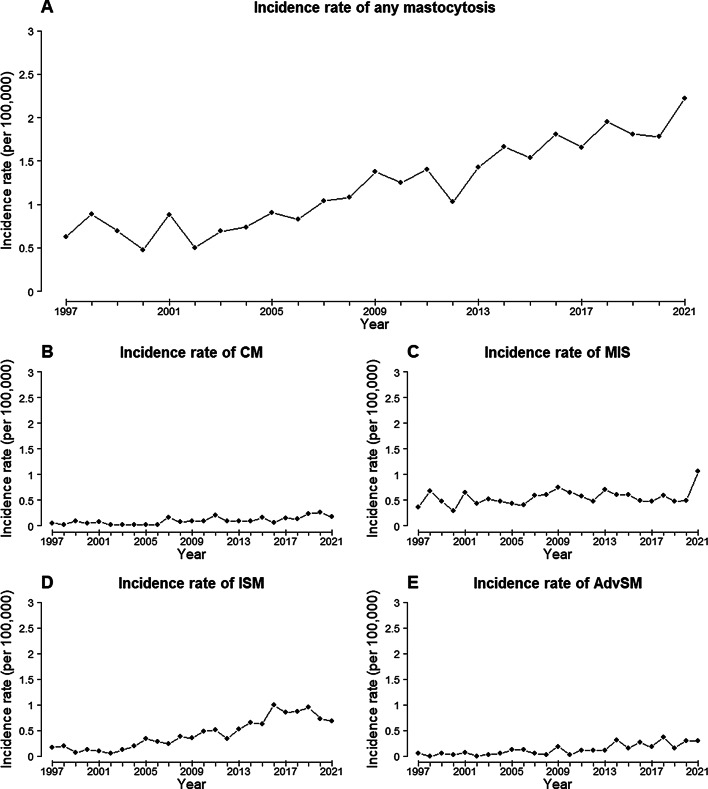
Yearly incidence rate of mastocytosis from 1997 to 2021 divided into **A**) all subtypes, **B**) cutaneous mastocytosis (CM), **C**) masto-cytosis in the skin (MIS), **D**) indolent systemic mastocytosis (ISM) and **E**) advanced systemic mastocytosis (AdvSM) comprised of aggressive systemic mastocytosis, systemic mastocytosis with an associated hematologic neoplasm and mast cell leukemia.

The prevalence of mastocytosis in Denmark as of January 1, 2022, was 27.43 per 100,000 (95% confidence interval (CI): 25.95–28.96). The prevalences of MIS and ISM were 13.94 per 100,000 persons (95% CI: 12.89–15.04) and 9.36 per 100,000 (95% CI: 8.51–10.28), respectively (Table 3). The average incidence rate of mastocytosis between 1997 and 2021 was 1.21 per 100,000 (95% CI: 1.02–1.40) (Table 3) with an increasing yearly incidence rate since 2002. The yearly incidence rate increased most among patients with ISM (Fig. 2).

**Table 3:** Prevalence (January 1st, 2022) and average incidence rate (1997–2021) of mastocytosis by subtype

Subtype	Prevalence (per 100.000) (95% CI)*	Average incidence rate (per 100.000 per year), mean (95% CI)
CM	2.29 (1.88–2.76)	0.10 (0.07–0.12)
MIS	13.94 (12.89–15.04)	0.55 (0.49–0.61)
ISM	9.36 (8.51–10.28)	0.43 (0.32–0.55)
ASM	0.13 (0.05–0.28)	0.01 (0.00-0.01)
SM-AHN	1.72 (1.36–2.13)	0.12 (0.07–0.16)
MCL	0	< 0.01 (0.00-0.01)
All subtypes	27.43 (25.95–28.96)	1.21 (1.02–1.40)

CI: Confidence Interval, CM: Cutaneous mastocytosis, MIS: mastocytosis in the skin, ISM: indolent systemic mastocytosis, ASM: aggressive systemic mastocytosis, SM-AHN: systemic mastocytosis with associated hematologic neoplasm, MCL: mast cell leukemia,

*Population in Denmark (age ≥ 18) on January 1st, 2022, was 4,721,691

### Survival

**Fig. 3:**
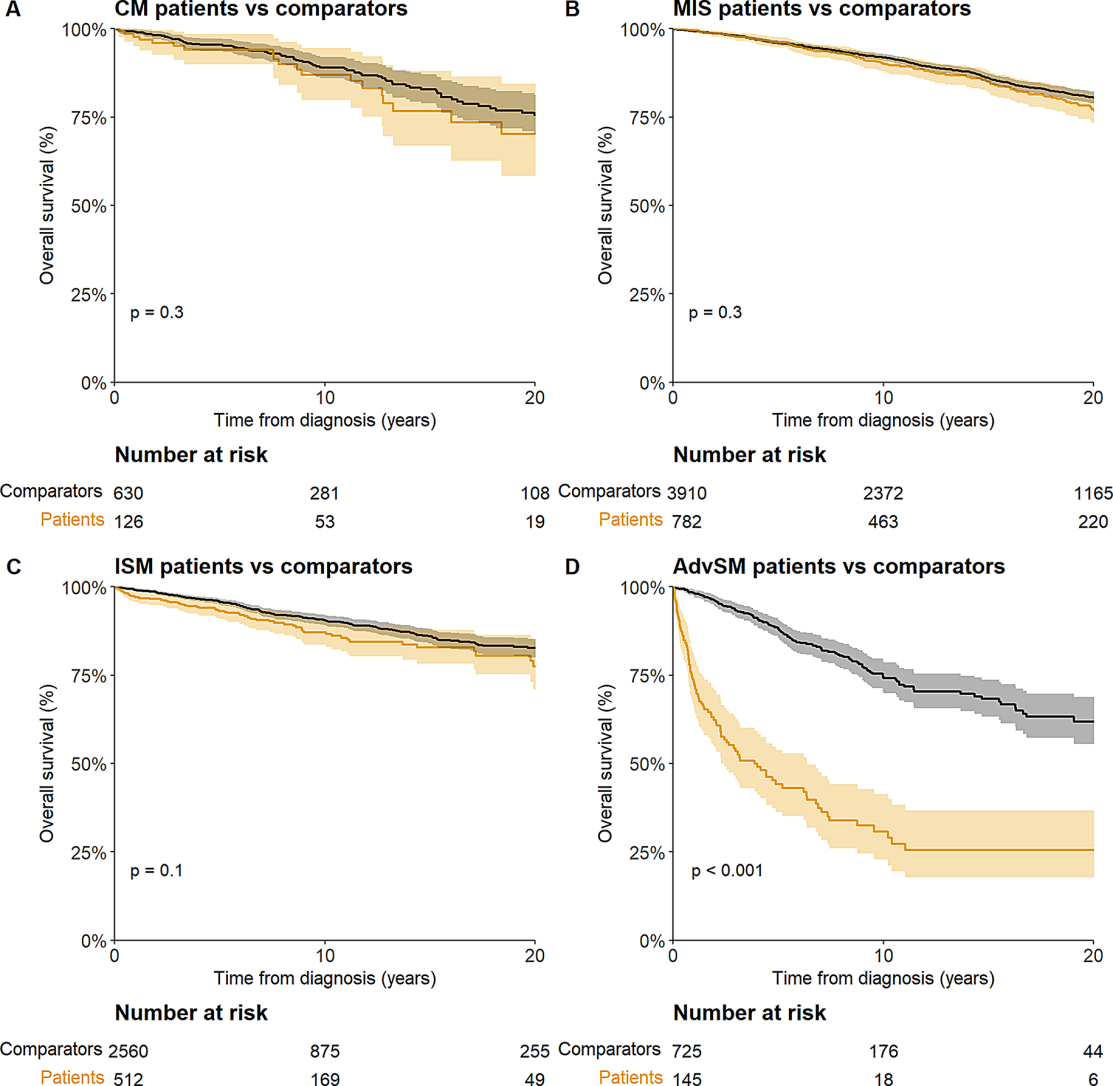
Overall survival of patients with **A**) cutaneous mastocytosis (CM), **B**) mastocytosis in the skin (MIS), **C**) indolent systemic mas-tocytosis (ISM) and **D**) advanced systemic mastocytosis (AdvSM) comprised of aggressive systemic mastocytosis, systemic mas-tocytosis with an associated hematologic neoplasm and mast cell leukemia. All patients were matched to 5 persons of the same sex and age from the general population. The p-value was constructed by testing for differences in restricted mean survival 20 years after diagnosis

Median follow-up was 11.3 years (95%CI: 10.8–11.5). Patients with AdvSM had a lower OS compared to their matched comparators (*p* < 0.001) whereas the OS of patients with CM, MIS and ISM did not differ from the general population (*p* = 0.3, *p* = 0.3, *p* = 0.1, respectively) (Fig. 3). The results did not change when dividing the patients according to the ICC classification (supplementary Fig. S2). The hazards of death for patients with CM and MIS were comparable to that for patients with ISM (ISM: HR 1.00 [reference], CM: HR 1.07 [95%CI: 0.55–2.08, *p* = 0.83], MIS: HR 0.96 [95% CI: 0.65–1.42, *p* = 0.84]), whereas the hazard of death for patients with AdvSM was considerably higher (HR: 4.40 [95% CI: 2.54–7.63, *p* < 0.001]) (supplementary Table S3). The 10-year survival probability was 86.8% (95%CI: 79.9–94.4%), 90.1% (95%CI: 87.8–92.5%), and 87.1% (95%CI: 83.6–90.8%) for CM, MIS, and ISM, respectively. The 10-year survival probability was 66.7% (95%CI: 37.9–100%) for patients with ASM, 29.8% (95%CI: 21.6–41.3%) for patients with SM-AHN and 0% for patients with MCL. The median survival was 3.1 years (95%CI: 2.3–6.2 years) for patients with SM-AHN and 0.3 years (95%CI: 0.1-NA) for patients with MCL.

## Discussion

By combining the Danish health registers, we have identified a large cohort of adult patients with mastocytosis with a long median follow-up of more than 11 years. The 1,594 patients represent all adult Danish patients diagnosed with mastocytosis prior to 2022. We found an overall female predominance among patients with mastocytosis, but a male overrepresentation among patients with AdvSM. The median age was 49 years although the patients with AdvSM were generally older. Both the age and sex distributions found in this study are comparable to previous findings [4, 15, 19, 39, 40].

In this study, we found a large proportion of patients with MIS, typically identified through skin biopsy. These patients were slightly younger and only a smaller portion of the patients were tested for KIT mutation or level of tryptase. Of the patients tested, 55.6% were positive for KIT mutation, and a lower level of tryptase was found compared to patients with ISM. This indicate that a full diagnostic examination was reserved to patients with more severe symptoms, elevated tryptase, and positive KIT mutation, and thereby a relative underrepresentation of this group of less symptomatic patients in the ISM group. The incomplete diagnostics are in line with the findings of Pyatilova et al., who found that most clinicians outside the Mastocytosis Centers of Excellence differs from the recommendation guidelines of diagnostics [41]. The Centers of Excellence in Europe and the United States of America were created in the early 2000s to centralize the treatment and create more knowledge on mastocytosis [20]. This has probably affected the diagnostics in Denmark, as it is evident that fewer patients were categorized as MIS during the latest period from 2017 i.e., 18.6% compared to 35% before 2001. Likewise, more patients have been categorized as either CM or ISM since 2017 i.e., 33.3% and 38.7% respectively compared with 25.1% and 10.2% before 2001 (Table 2). Thereby indicating a growing awareness on mastocytosis after the establishment of the Center of Excellence in Denmark in 2010 [20] and more focus on the appropriate diagnostics of this patient group. Most MIS patients identified in this study will undoubtedly have non-advanced SM, making ISM by far the most common subtype of mastocytosis as previously found [5, 15, 18, 42, 43]. Furthermore, the percentage of adult patients with CM found in this study is higher than previously found i.e., 8.3% in this study and 4% in the study by Zanotti et al. [18]. Some of the patients had a positive KIT mutation (42.9%) and a high tryptase level which is not to be expected and could be a sign of undiagnosed SM. An explanation could be the at times loosely scattered pattern of abnormal mast cell in the BM making correct diagnosis difficult [8] as well as a previously lower sensitivity of the diagnostic methods. If these patients were examined today, it would be reasonable to assume that some would be diagnosed with SM, most likely ISM.

In this study, 3.6% of patients with an initial diagnosis of non-advanced mastocytosis (MIS or ISM) progressed to an AdvSM after a median of 7 years with the majority progressing from ISM. Furthermore, contrary to the findings of Kluin-Nelemans et al. we found a slight majority of women among those who progressed [39]. The frequency of progression in this study is higher than the findings of Trizuljak et al., in which 2.9% of patients with non-advanced SM progressed to AdvSM during a median follow-up of 4.2 years [44] and the findings of Zanotti et al. in which 1.9% progressed from indolent mastocytosis to AdvSM during a median follow-up of 6 years [18]. In comparison, the study by Ungerstedt et al. did not observe any progression during a median of 7.5 years follow-up [5]. The higher percentages of progression found in this study could be due to the longer follow-up time as the median of 7 years from initial diagnosis to progression highlight the necessity of long-term follow-up.

The prevalence of mastocytosis has risen during the last two decades since van Doormal et al. and Cohen et al. found a prevalence of approximately 10–13 per 100,000 persons in 2013–2014 [16, 17]. In this study, the prevalence of mastocytosis was 27.43 per 100,000 persons. This is higher than previous studies, yet comparable to the recent finding of Bergström et al. who found a prevalence of mastocytosis of 23.9 per 100,000 persons among patients aged 20 years or more from 2001 to 2018 in Sweden [19]. However, in the Swedish study more than 350 patients with a diagnosis of D47.0 were included. In our study, these patients were excluded as a diagnosis of mastocytosis could not be validated. Furthermore, as different age restrictions and time periods were imposed, this might also influence the difference in the prevalence found. Caution should therefore be taken when comparing the findings in this study to previous epidemiologic studies as they are quite heterogeneous with different age groups and subtypes of mastocytosis being examined. The combined prevalence of SM found in this study was 11.19 per 100,000 persons which is comparable to the finding of Zanotti et al. (10.2 per 100,000) and Ungerstedt et al. (10.6 per 100,000) in the Veneto and Stockholm region, respectively [5, 18]. However, the large proportion of MIS patients identified in this study will inevitably disturb this similarity.

We found an average incidence rate of 1.21 for any type of mastocytosis and 0.56 for SM patients with increasing incidence rate since 2002. These numbers are slightly lower than the incidence rates found by Bergström et al. [19] and Ungerstedt et al. [5]. The average incidence rates found in this study are highly affected by previous years with lower incidence and would be higher if only the last decade had been evaluated. Accordingly, a higher incidence of SM was seen in the study by Zanotti et al. as only the incidence since 2018 was evaluated [18].

In this study, we confirmed earlier findings of a favorable prognosis of non-advanced mastocytosis comparable to the general population, and an overall poor survival in the AdvSM group [15]. Furthermore, we found no differences in death rates among patients with CM, MIS, and ISM. Among patients with AdvSM the prognosis was considerably worse for patients with MCL with a median survival of only 0.3 years. A better OS was seen among patients with ASM with 66.7% being alive 10 years after the diagnosis. However, for both MCL and ASM the results must be evaluated carefully as they are based on very small groups. A study by Kluin-Nelemans et al. found that across all subtypes of mastocytosis male patients had poorer survival compared to female patients [39]. This aspect was not investigated in the current study; however, further study into survival differences across the sexes compared to the general population would be relevant.

There are several strengths to this study. The most important being the Danish high-quality health registers which allow for identification of all patients diagnosed with mastocytosis and enable thorough and longtime follow-op. By combining SNOMED codes for morphology and topography with ICD codes based on diagnoses made in specialist care, we ensure high validity of the cohort and minimize selection bias. There are some limitations of the study. Using the registers there is a risk of misclassification of the subtypes as it was not possible to identify factors usually used to differentiate between non-advanced and AdvSM e.g., mast cell burden, organ dysfunction, organomegaly, cytopenia, or weight loss. Thereby, patients with ASM could possibly be underreported as we relied on the specific SNOMED and ICD-10 codes registered. Furthermore, the use of the registers could also influence the number of patients classified with progression as a later registration of a more severe subtype of mastocytosis could be due to disease clarification instead of progression. However, as most patients who progressed developed a biopsy-verified AHN this is not believed to have influenced the results significantly. Moreover, the choice to divide the patients according to the WHO classification instead of the ICC classification increased the number of progressions; yet this was done in order to compare to previous studies in which the WHO classification was used. Another limitation is the rarity of mastocytosis as it can be difficult for the general practitioner to recognize the signs and symptoms of the disease, leaving some patients undetected, and the prevalence found in this study would thus be underestimated to an unknown degree.

In conclusion, the prevalence of mastocytosis among adults in the Danish population is higher than previously found which is expectable with the general good survival among the majority of the patients. However, an increasing incidence rate reveal, that more and more patients are being diagnosed each year. Thereby indicating that more awareness of the disease since the establishment of the Mastocytosis Centers of Excellence combined with better diagnostic methods have led to more cases of mastocytosis being detected.

## Electronic supplementary material

Below is the link to the electronic supplementary material.

Supplementary Material 1

## Data Availability

The project was carried out on a highly secured server at Statistics Denmark, for which only a limited number of scientists are allowed access to ensure the safety of the data. Research data are therefore not shared, as export of data from Statistics Denmark is not allowed.
