# Supplementary material for: Prevalence and incidence of mastocytosis in adults: a Danish nationwide register study
Source: Eur J Epidemiol. 2025 Jan 3;40(1):43–53. doi: 10.1007/s10654-024-01195-5 (PMC11799073; doi:10.1007/s10654-024-01195-5)
Supplement: Supplementary file 1 — Supplementary Material 1 [file 10654_2024_1195_MOESM1_ESM.docx]

**SUPPLEMENTARY**

Table S1 – Identification of registered patients

|  | ICD-8 | ICD-10 | SNOMED |
| --- | --- | --- | --- |
| CM | 75722 | Q82.2, Q82.2A, Q82.2B, D47.0, D47.0A, D47.0B | Morphology: M97411, S35600.  Topography: *T01-T03*  +  Normal findings in BM biopsy (T06) |
| MIS |  | Q82.2, Q82.2A, Q82.2B, D47.0, D47.0A, D47.0B | Morphology: M97411, S35600.  Topography: *T01-T03*  No biopsy performed on BM (T06) |
| ISM |  | D47.0, D47.0A, D47.0B, Q82.2 | Morphology: M97411, M974A1, S35650.  Topography: *T06, T07-T09, T10-T11, T26-T29, T56-59, T60-T69, T74-T75 and/or TX1-TX7* |
| ASM |  | C96.2 | Morphology: M97412, M97413, M97417, M97418, M97403. Topography: *T06, T07-T09, T10-T11, T26-T29, T56-59, T60-T69, T74-T75 and/or TX1-TX7* |
| SM-AHN |  | SM:  D47.0, D47.0A, D47.0B, D47.0C, D47.0B, Q82.2, C96.2, C94.3  +  AHN:  C81-C86, C88, C90-C96 (*except C94.3, C96.2*), D45, D46, D47.1, D47.3, D47.4 | SM:  Morphology: M97411, M974A1, S35650, M97412, M97413, M97417, M97418, M97403, M97422, M97423, M97427, M97428S.  Topography: *T06, T07-T09, T10-T11, T26-T29, T56-59, T60-T69, T74-T75 and/or TX1-TX7*  +  AHN:  M959, M960, M961, M962, M963, M964, M965, M966, M967, M968, M969, M970, M971, M972, M973, M974, M975, M976, M977, M978, M979, M980, M981, M982, M983, M984, M985, M986, M987, M988, M989, M990, M991, M992, M993, M994, M995, M996, M997, M998, M999   - Except SNOMED codes for mastocytosis - Except OBS PRO SNOMED codes |
| MCL |  | C94.3 | Morphology: M97422, M97423, M97427, M97428.  Topography: *T06, T07-T09, T10-T11, T26-T29, T56-59, T60-T69, T74-T75 and/or TX1-TX7* |

CM: Cutaneous mastocytosis, MIS: mastocytosis in the skin, ISM: indolent systemic mastocytosis, ASM: aggressive systemic mastocytosis, SM-AHN: systemic mastocytosis with associated hematologic neoplasm, MCL: mast cell leukemia

Morphology and topography coded to the same date.

Table S2 – Differences in age at initial diagnosis, sex and tryptase levels near the initial diagnosis of patients initially diagnosed with ISM or MIS but divided according to progression.

| Characteristic | ISM,  N = 488 | Progressed from ISM, N = 29 | p-value | MIS,  N = 780 | Progressed from MIS, N = 19 | p-value |
| --- | --- | --- | --- | --- | --- | --- |
| Age, median (IQR) | 49 (40-63) | 56 (46-70) | 0.012 | 45 (32-58) | 59 (46-64) | 0.008 |
| Sex, n(%) |  |  | 0.2 |  |  | 0.027 |
| Female | 263 (53.9%) | 19 (65.5%) |  | 483 (61.9%) | 7 (36.8%) |  |
| Male | 224 (46.1%) | 10 (34.5%) |  | 297 (38.1%) | 12 (63.2%) |  |
| Initial tryptase level (ng/ml), median (IQR) | 22 (11-52) | 49 (40-71) | 0.04 | - | - | - |
| Missing information | 53 | 21 |  | - | - | - |

ISM: indolent systemic mastocytosis, MIS: mastocytosis is the skin, IQR: Interquartile range.

Progression defined as >180 days from initial diagnosis. Initial tryptase level defined as the registered result closest to the initial diagnosis date.

Table S3 – Comparison of the hazards of death between subgroups

| Type | HR (95% CI) | p-value |
| --- | --- | --- |
| ISM | 1.00 (reference) | - |
| CM | 1.07 (0.55-2.08) | 0.83 |
| MIS | 0.96 (0.65-1.42) | 0.84 |
| AdvSM | 4.40(2.54-7.63) | <0.001 |

HR: Hazard ratio, ISM: indolent systemic mastocytosis, CM: Cutaneous mastocytosis, MIS: mastocytosis in the skin, AdvSM: advanced systemic mastocytosis (aggressive systemic mastocytosis, systemic mastocytosis with an associated hematologic neoplasm, mast cell leukemia). Cox proportional hazard model using ISM as reference and adjusted for sex, age at initial diagnosis and year of initial diagnosis.

Figure S1 - Overall survival of adult patients mastocytosis compared to matched comparators.


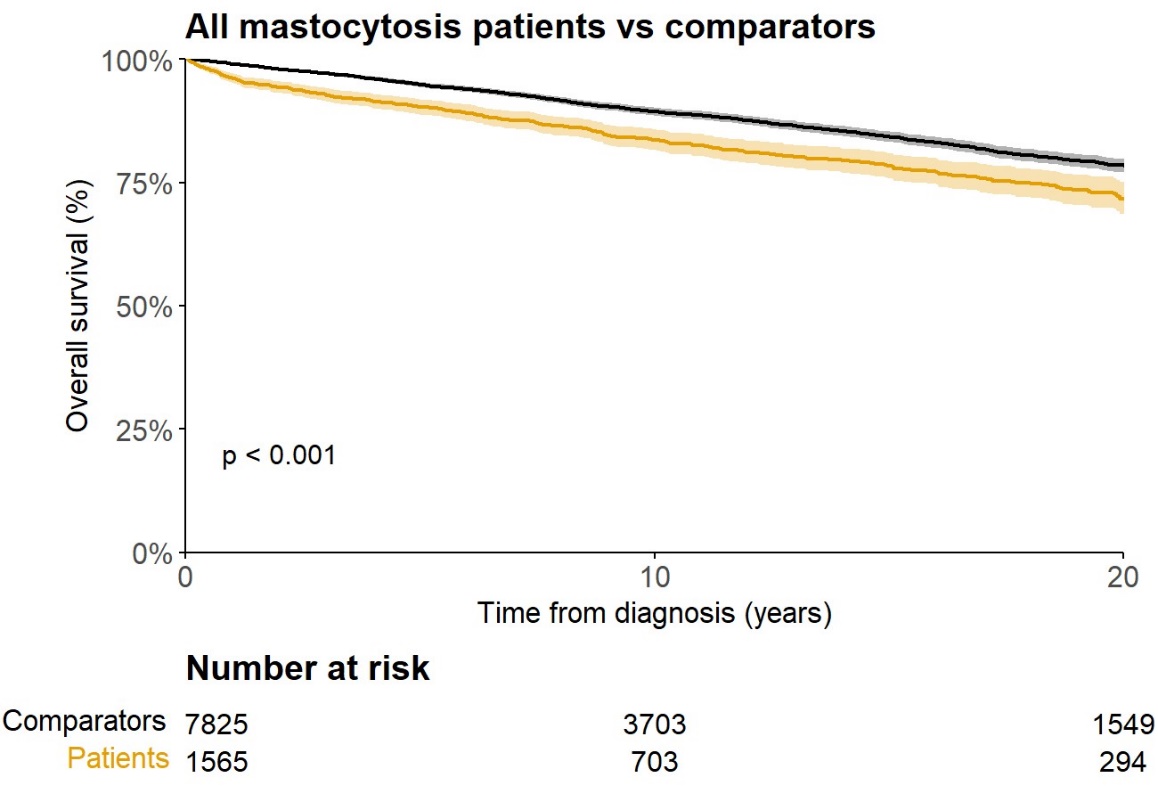
*Figure S1: Overall survival of patients with any type of mastocytosis. All patients were matched to 5 persons of the same sex and age from the general population. The p-value was constructed by testing for differences in restricted mean survival 20 years after diagnosis.*

Figure S2 – Overall survival of patients categorized using the ICC classification


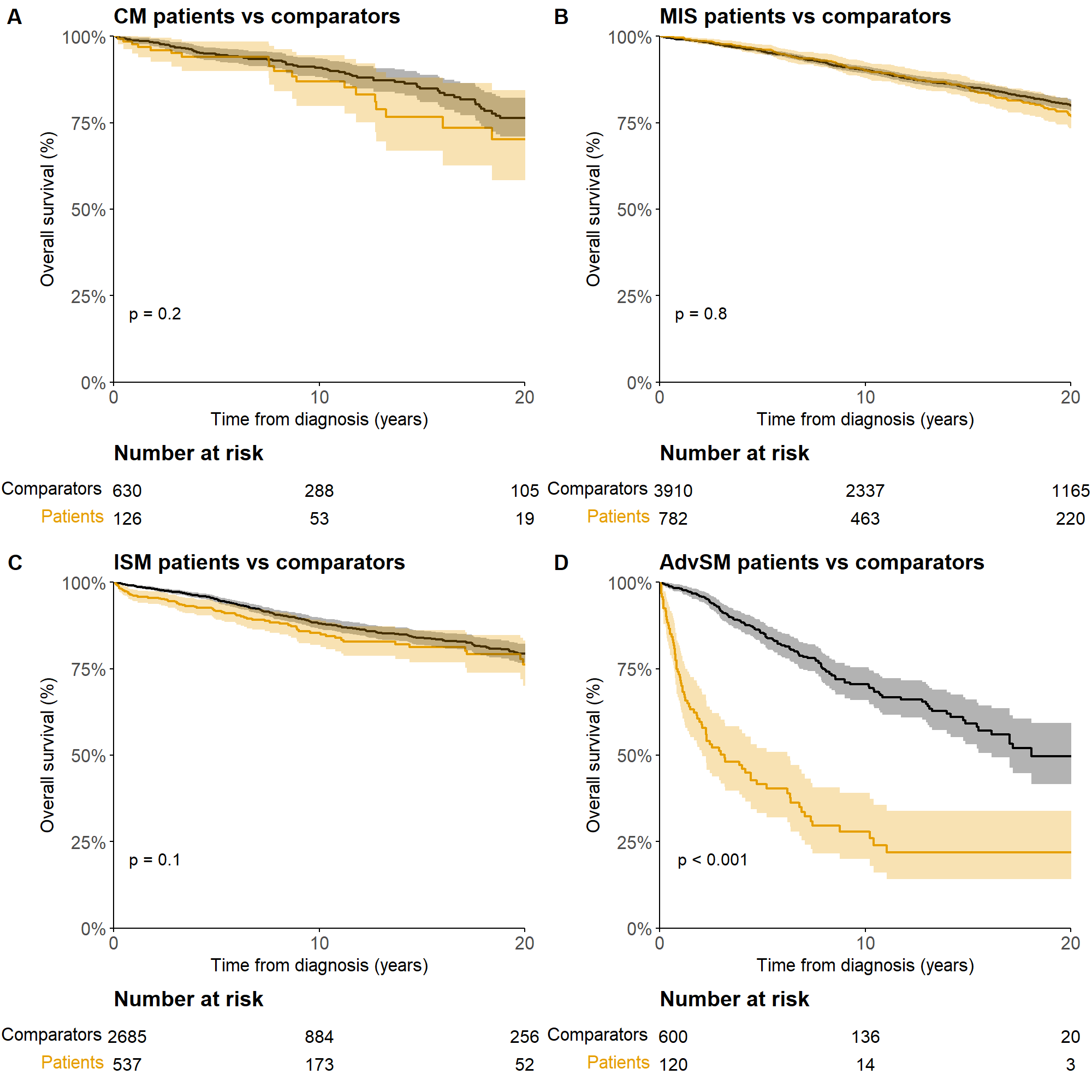


*Figure S2: Overall survival of patients with A) cutaneous mastocytosis (CM), B) mastocytosis in the skin (MIS), C) indolent systemic mastocytosis (ISM) and D) advanced systemic mastocytosis (AdvSM) comprised of aggressive systemic mastocytosis, systemic mastocytosis with an associated myeloid neoplasm and mast cell leukemia. All patients were matched to 5 persons of the same sex and* *age from the general population. The p-value was constructed by testing for differences in restricted mean survival 20 years after diagnosis.*
